# Untimely illness: When diagnosis does not match age‐related expectations

**DOI:** 10.1111/hex.12669

**Published:** 2018-02-09

**Authors:** Susan Kirkpatrick, Louise Locock, Albert Farre, Sara Ryan, Helen Salisbury, Janet E. McDonagh

**Affiliations:** ^1^ Nuffield Department of Primary Health Care Sciences University of Oxford Oxford UK; ^2^ Health Services Research Unit University of Aberdeen Aberdeen UK; ^3^ Institute of Applied Health Research University of Birmingham Birmingham UK; ^4^ Centre for Musculoskeletal Research University of Manchester Manchester UK

**Keywords:** adult‐onset asthma, age and diagnosis, age‐related expectations, candidacy, chronic illness, diagnosis, diagnostic delay, young people with arthritis

## Abstract

**Background:**

We explore the concept of “untimely diagnosis,” where the onset of a long‐term condition occurs at a life stage which does not conform to traditional expectations, focusing on two conditions (asthma and arthritis) typically associated with a particular life stage (childhood and older adulthood, respectively). Previous literature has focused on the meaning of chronic illness in terms of life history, and the biographical lens has been used in various ways to make sense of the experience. Less attention has been paid to the condition onset when it seems dissonant with chronological age.

**Methods:**

Secondary analysis of two qualitative data sets (total 58 interviews) exploring the experiences of people with adult‐onset asthma and young people diagnosed with arthritis. Data from the original interview transcripts relating to diagnosis and symptom recognition were re‐analysed using a “candidacy” framework to examine how age and diagnosis intersect.

**Results:**

People did not always assert their candidacy for either condition because of pre‐conceived expectations around age. Similarly, health professionals sometimes failed to recognize patients’ candidacy, instead pursuing “age‐plausible” possibilities. In some cases, participants were proactive in suggesting a diagnosis to the health professional where diagnosis was delayed.

**Conclusion:**

The diagnosis of adult‐onset asthma, and arthritis in young people, may be regarded as “untimely.” We suggest that being diagnosed with what is perceived to be a “childhood” condition in adulthood, or “an older person's” condition in childhood, may be viewed as a “biographical paradox” and an “untimely breach” to the expected order.

## 1 INTRODUCTION

There has been a growing interest in the sociology of diagnosis in recent years. Here, we focus on narratives of asthma in adulthood, and arthritis in young people, to explore the idea of “untimely diagnosis,” where a diagnosis occurs at a life stage which does not conform to individual or societal expectations of that condition.

Diagnosis is increasingly recognized as a process involving a series of interactions with health‐care systems and staff, rather than strictly as a “diagnostic moment” at which the health professional communicates a medical label to a patient.^1^, ^2^ The individual and their family also play a key role in puzzling out a diagnosis, seeking to make sense of symptoms and clues from health professionals’ behaviour, and drawing on their common sense stock of knowledge.^3^, ^4^

…diagnosis provides a cultural expression of what society is prepared to accept as normal and what it feels should be treated…. [It] is an important site of contest and compromise, because it is a relational process with different parties confronting illness with different explanations, understandings, values and beliefs. (p. 291)

Of relevance here is the concept of “candidacy,” redefined by Dixon‐Woods et al's^5^ critical synthesis as “the ways in which people's eligibility for medical attention and intervention is jointly negotiated between individuals and health services.” The focus of the critical synthesis was access to health care by vulnerable groups, and the authors found that candidacy is a continually negotiated property of people, subject to various influences including social contexts, aspects of self, situated activity and resource allocation. The core strands of the new theoretical conceptualization of access to health care are as follows: the identification of candidacy, navigation (of services and support), the permeability of services (the ease with which people can use them), appearance, adjudication, resistance and local conditions. Three factors are identified that contribute to the downgrading of warning signs of illness for vulnerable groups: the lack of a positive conceptualization of health; the normalization of symptoms within deprived communities; and the fear of being blamed by health professionals.

This work has been extended by various studies including MacDonald et al^6^ who found hazy and indistinct boundaries between these components of candidacy. Their paper further highlights how age and gender can compromise candidacy and the authors conclude “It is important that we do not underestimate the ways in which our shared understandings of the signs of illness influences patients’ experiences of care, and importantly, access to care” (p. 109).

Davison et al,^7^ who introduced the notion of candidacy, remind us that we “tend not to invent completely fresh explanations [for the misfortunes which befall ourselves or other people]; rather we employ the knowledge and lore which we have received from the wider society during our formation and development as individuals” (p. 5).

In this study, we explore how people experience and make sense of what both they themselves, and health professionals with whom they interact, may perceive as an “untimely diagnosis.” We use the candidacy framework to consider how knowledge and lore may mask a “curveball” such as an untimely diagnosis and disrupt typical help‐seeking behaviour. We explore what may happen when possible diagnoses seem to defy the “common sense” stock of knowledge by occurring at a time of life which does not “fit,” using adult‐onset asthma and arthritis in young people as exemplars.

We considered the candidacy framework to be more appropriate than, for example, the theory of illness representations^8^ which more commonly focuses on people's beliefs and expectations about their illness after diagnosis. However, Bishop and Converse's study^9^ of how people invoke “prototyped conceptions” to make sense of a particular set of symptoms is an interesting use of illness representations relevant to this pre‐diagnostic phase.

### 1.1 The importance of age in making sense of chronic illness

A diagnosis of long‐term illness is unlikely to be welcome at any stage of life but may still be understood as part of a “normal” life trajectory. Following Bury's original exploration of chronic illness as biographical disruption,^10^, ^11^ it has been argued that it may instead be accepted as biographically anticipated “normal illness,” and has been described using terms such as “biographical flow,” “biographical anticipation” or “biographical continuity”.^11^ The meaning of chronic illness to people has been found to be dependent partly on their age. Thus, contrasting the perspectives of young people and older adults provides a particularly good exemplar to illustrate the importance of age as a mediating factor in the experience of chronic illness.

Sanders, Donovan and Dieppe^12^ for example, found that older people interpreted the pain and impaired mobility of osteoarthritis as a normal part of ageing, consistent with their expected biography rather than disruptive of it (even if at the same time it disrupted practical daily living). In explaining their findings, they drew on Bury's^13^ distinction between “meanings as significance” (in this case, the significance—or not—of the condition for one's sense of self) and “meaning as consequence” (in this case, the practical activity restriction and social disadvantage resulting from the condition).

The meaning of illness may also depend on comorbidity and wider social factors such as class, poverty and poor housing, as well as the type of illness and social perceptions of it. Faircloth et al^14^ in a study of experiences of stroke amongst white, Hispanic and African Americans argue that their findings “suggest a biographical flow more than a biographical disruption to specific chronic illnesses once certain social indicators such as age, other health concerns and previous knowledge of the illness experience, are taken into account” (p. 242).

Pound et al,^15^ in a study of older working class people in London, suggest they may have “lower expectations of health and may anticipate illness as inevitable in old age, or meet it with a greater sense of acceptance” (p. 502). The participants in this study described witnessing death at a relatively early age amongst family and friends, alongside experience of other hardships. In this context, Pound et al encourage us to pay attention to “the straightforward possibility offered by the interviewees themselves” that “chronic illness may be anticipated and experienced by some older people as normal” (p. 502).

Bury and Holme recognized this as a valuable qualification of the original idea, as they point out, most people operate a “social clock” that guides expectations of events.^16^ As Williams^11^ describes it:

Prejudging the issue of illness as biographical disruption cannot, from this viewpoint, be justified. Instead, timing and context, norms and expectations, alongside our commitment to events, anticipated or otherwise, are crucial to the experience of our lives, healthy or sick, and the meanings with which we endow it. (p. 51)

When it comes to the experiences of young people, the relevance of “biographical disruption” cannot be taken for granted either. This is particularly true in cases of congenital illness, such as cystic fibrosis, where there is no prior period of wellness or perceived normality.^17^ Similarly, the relevance of “biographical disruption” is also unclear when the experiences and meanings of chronic illness take into account that a major task of adolescent development, healthy or otherwise, is the development of one's identity or “self‐concept”.^18^ In this context, chronic illness can be seen more as “biographical contingency” than as “biographical disruption”,^19^ with young people attempting to reconcile their pre‐illness identity with developing an altered identity that incorporates chronic illness as an accepted component of life.^20^ However, other researchers such as Grinyer^21^ have suggested that adolescent development in fact exacerbates biographical disruption, given that at this stage, identity is particularly fragile and key developmental goals such as increased autonomy and independence could be compromised.

Age is thus clearly one major factor affecting the likelihood that a diagnosis is seen as normal or expected, and much of the literature has focused on examples where illness is congruent with age‐related expectations. There has been less attention paid to situations where the onset of chronic illness is seen as dissonant with chronological age. For example, it is important to acknowledge the inherent difficulty young people with chronic conditions face in navigating the fundamental tension between the very concept of youth, typically portrayed as a time of health, and being diagnosed with a chronic illness at a young age.^22^, ^23^

### 1.2 Chronic illness as an age‐dissonant life event: adult‐onset asthma and young people with arthritis

Some conditions are commonly associated in the popular imagination with particular life phases. This may include “childhood” infections such as chickenpox or measles; hypertension as a condition of older adults; type 1 diabetes as a condition of childhood or adolescence, contrasted with type 2 typically associated with late middle age; or dementia as a condition of old age. Popular representations in the media and online images for diseases such as these tend to reinforce these understandings. In all these cases, however, the condition may be diagnosed at a different and unexpected time of life. For example, Khanolkar et al^24^ note increases in diagnoses of type 2 diabetes in adolescence particularly in ethnic minority populations. Similarly, Higginbottom^25^ found that being diagnosed with hypertension at a young age was perceived to be stigmatizing for young people, with hypertension commonly assumed to be a condition that only older people experience as part of the ageing process.

In this study, we focus on two conditions that are closely associated with a particular time of life: asthma, which is commonly thought of as a disease of children and young people, and arthritis, which is typically represented as a disease of older adulthood.^26^

In both cases, we explore the perspectives of those whose diagnosis does not match such age‐related expectations

*Asthma* is a chronic and potentially life‐threatening disease characterized by recurrent attacks of breathlessness and wheezing which currently is estimated to affect 335 million people worldwide and is the most common chronic disease amongst children.^27^ In children and young people, asthma is most often associated with allergies (atopic asthma). Asthma that comes on later in life is less obviously allergic and is more common in women and smokers. A number of risk factors that may be associated with the onset of asthma in adulthood have been recognized, including respiratory infections, environmental factors, hormones, obesity and stress.^28^ The diagnosis of asthma in older adults can present something of a challenge for various reasons. Many older people, particularly with late‐onset asthma, fail to identify breathing difficulties as asthma initially.^29^ They, or their doctor, may first attribute symptoms to other causes such as bronchitis, respiratory tract infection, cardiac disease, ageing or lack of fitness.^30^, ^31^, ^32^

*Arthritis* is an umbrella term covering joint pain and inflammation arising from a range of causes. In popular discourse, “arthritis” is often taken to mean osteoarthritis, and in turn considered to be age‐related wear and tear of the cartilage in joints which results in painful rubbing of bone on bone; although recent research is revealing a more complex condition now considered to be a disease characterized by “tear, flare and repair”.^33^ In young people, however, arthritis is more likely to be a form of inflammatory arthritis, which is an autoimmune condition.

Young people with inflammatory arthritis live with chronic or recurrent pain and disability, particularly if there has been a delay in starting effective treatment, which can limit their ability to complete daily physical tasks and participate in school and social activities.^32^, ^34^, ^35^ In over a third of young people, the disease remains active into adulthood requiring drug therapy.^36^

The diagnosis depends on the demonstration of clinical signs, rather than laboratory tests, with no other defined diagnosis being evident.^37^ In the UK, all patients both children and adults present with their symptoms to a General Practitioner (family doctor) in the first instance. For patients where an inflammatory arthritis is suspected, GPs should refer to a specialist rheumatologist to confirm the diagnosis and initiate disease‐modifying treatment. Treatment is often complex and challenging and involves ongoing medications, monitoring, physical therapy and surgery in some cases.^34^, ^35^

## 2 METHODS

We used secondary analysis methods to explore how people make sense of a diagnosis of a condition that may not “fit” with popular expectations, particularly in relation to age. Our secondary analysis involved two data sets from two separate primary studies. Both primary studies were qualitative, narrative interview studies, addressing the following research question: “what are the experiences and information and support needs of people with a (particular) health condition?^38^ Both were conducted using the overarching methods and guidelines developed and refined by [Health Experiences Research Group, University of Oxford]. The studies have approval from [NRES Committee South Central – Berkshire Ref 12/SC/0495 IRAS ID 112111] which includes a licence agreement for the interviews to be shared with other universities and used for further research, including secondary analysis of the transcripts for additional publications.

Both studies used a purposive maximum variation sampling approach^39^ to include variation across types of experience (such as time since diagnosis and degree of disease severity/progression) and demographic variables (such as gender, age, ethnicity, socio‐economic group and region). A range of recruitment avenues were used including through GPs, specialist nurses and hospital clinics; voluntary support groups; media advertising; word of mouth and snowballing.

Our secondary analysis was based on a total of 58 interviews, including a subset of 18 people diagnosed with adult‐onset asthma from the first primary study (Table 1); and 40 people diagnosed with arthritis in childhood or adolescence and 9 carers from the second primary study (Table 2).

**Table 1:** Subset of participants diagnosed with adult‐onset asthma from the first primary study

Participants	Age at interview	Age at diagnosis	Number of years living with the condition	Gender
1	59	18	41	Female
2	34	18	16	Female
3	63	25	38	Male
4	25	25	0	Male
5	52	23	29	Female
6	41	34	7	Female
7	68	40	28	Female
8	62	45	17	Male
9	55	40	15	Female
10	54	36	18	Female
11	71	40	31	Male
12	62	47	15	Female
13	48	33	15	Female
14	62	42	20	Female
15	62	57	5	Female
16	55	53	2	Female
17	59	54	5	Female
18	69	53	16	Male

**Table 2:** Participants diagnosed with arthritis in childhood/adolescence and carers from the second primary study

Participants	Age at interview	Age at diagnosis	Number of years living with the condition	Gender
Young people				
1	21	21	2 mo	Male
2	25	22	3	Female
3	12	8	4	Male
4	14	10	4	Female
5	14	12	2	Male
6	17	16	1	Female
7	16	6	10	Male
8	24	4	20	Female
9	14	12	2	Male
10	20	1	19	Female
11	25	17	8	Female
12	13	13	4 mo	Female
13	15	1	14	Female
14	18	14	4	Male
15	28	2	26	Male
16	13	4	9	Female
17	16	11	5	Female
18	20	1	19	Female
19	23	18	5	Female
20	22	20	2	Female
21	21	9	12	Female
22	14	11	3	Female
23	18	1	17	Female
24	26	13	13	Female
25	20	18	2	Female
26	16	11	5	Female
27	19	13	6	Female
28	10	7	3	Female
29	26	2	24	Female
30	15	11	4	Female
31	24	14	10	Female
32	40	12	28	Female
33	19	13	6	Male
34	26	3 mo	26	Male
35	17	10	7	Male
36	22	11	11	Female
37	25	13	12	Female
38	22	5	17	Female
39	28	5	23	Female
40	21	12	9	Female
Carers				
1	33	N/A	N/A	Female
2	40	34	6	Female
3	42	N/A	N/A	Female
4	47	N/A	N/A	Female
5	55	N/A	N/A	Female
6	39	N/A	N/A	Female
7	54	N/A	N/A	Male
8	43	N/A	N/A	Female
9	38	N/A	N/A	Female

Interviews for each typically lasted between 1 and 2 hours. They were conducted in the participants’ own home or elsewhere if they preferred and were video or audio‐recorded. In both studies, interviews started with an open‐ended invitation to tell the story of the individual's experience. Following this unstructured narrative, semi‐structured prompting was used to elaborate further. The topic guide in each case included many consistent areas (eg initial symptoms; path to diagnosis; referral and secondary care experiences; treatment; living with the condition; information and support needs) but also condition‐specific prompts derived from the literature and advice from an expert panel including patient representatives.

Consent was sought on the day for the initial interview; participants were later sent a verbatim transcript of their interview to review before final consent and copyright was agreed for publication of extracts from the data online. The initial analysis of both studies involved the use of NVivo software for coding the data and thematic analysis drawing on grounded theory techniques of constant comparison and deviant case analysis.^40^

In both studies, a second researcher provided an additional critical perspective and layer of rigour by independently analysing coding reports and comparing and discussing [their] interpretation with that of the original researcher. Summarized findings of the full thematic analysis of each study are available online [at http://www.healthtalk.org ].

The secondary analysis for this study came about because the authors were struck by the ways in which participants with both conditions spoke about their diagnosis in relation to age. Two of the authors [SK and LL] were involved in the original projects and were therefore well placed to contextualize the material in the light of the full interview material.

For this secondary analysis, the full transcripts were re‐coded by [anonymized for peer review] to identify all material related to diagnosis, symptom recognition and referral, and re‐analysed using a “candidacy lens.” Particular attention was paid to the ways in which age‐related expectations arose within the narratives. Because the secondary analysis was undertaken by two of the original researchers, we were confident that relevant data extracts were included. A series of workshop meetings in which the re‐coded data were discussed and reviewed jointly by the authorship group added a further layer of scrutiny and provided opportunities to debate the emerging findings. This process falls within Heaton's categories of amplified and supplementary analysis.^41^

### 2.1 Findings

Both conditions were typically seen as “belonging” to a certain age or stage of life. Stereotypical images of arthritis as a condition typically affecting older people were frequently invoked:

You know, you just thought of old people with it […] you don't think you're going to be using a Zimmer frame when you're ten years old.

(James, diagnosed age 10)

I remember in school learning about arthritis a little bit in biology and you always, you always assume it's something that older people get in their old age and never attribute it to someone your age. […] I just thought, it couldn't happen because I'm far too young and I'm just, I was in this denial for a while.

(Daniel, diagnosed age 21)

Conversely, asthma was commonly perceived as a childhood condition and many expressed surprise later when they discovered that it could start in adulthood:

I just assumed people got it as young children and kept it or got rid of it….. I hadn't realised that you could be diagnosed as an adult with it.

(Veronica, diagnosed age 57)

Asthma wasn't in my consciousness because I thought it was something children had and grew out of, I'd never heard of asthma appearing in your life when you were 40.

(Jackie, diagnosed age 40)

#### 2.1.1 Delaying help‐seeking—missed candidacy

These commonly perceived ideas about each condition played a key role in decisions about seeking medical attention. Participants themselves typically tended to play down their symptoms, or attribute them to a range of other causes.

Young people with arthritis commonly described accidents or circumstances that could be typically seen as “normal” in the context of the life and daily activities of a young person, such as having “broken my fingers maybe and not realised” (Kim, diagnosed age 22), sports injuries or, like Daniel, who said “I just thought it was growing pains or whatever, so I just ignored it” (Daniel, diagnosed age 21). Parents too would tend to make sense of initial symptoms by attributing them to more age‐plausible explanations.

Tessa remembers her daughter Lisa having problems with pain in her neck 6 months before she was diagnosed such that, when Lisa was in the car, sudden movements would hurt her neck. Initially though, she put this down to too much time on a computer game:

You see she'd got a little Nintendo DS from Santa and this was January so it was just after Santa came and we sort of put it down to the fact that she had her head down all the time. So I didn't think much of it I suppose. Children get sore necks and any bits of aches and pains all the time.

(Tessa, parent of Lisa diagnosed age 9)

Similarly, people often attributed the initial signs of asthma such as breathlessness, to other possible causes such as ageing, exercise or environmental factors:

I just felt as if my batteries had run out. I didn't feel that I was, well I was obviously breathless because I'd been running but I didn't feel that I was more breathless than I should have been, no other than usual.

(Jill, diagnosed age 52)

I think I just was a bit run down and generally struggling health wise, […]. We lived very close to a lot of pine trees on the south coast of England and I thought that the quality of the atmosphere and the hot summer might have been, the hot spring might have been a, [er] the cause.

(Debbi, diagnosed age 23)

Even where there was a family history of each condition, people still tended not to make the connection between what they had seen in other family members, and their own symptoms. For example, Jill (above) had not initially thought of asthma as an explanation for her breathlessness, despite explaining later in her story that “My son's had asthma since a child.” Similarly, Todd's sister had severe asthma as a child and had died from it, but although he had sometimes used her asthma medication to alleviate his own breathlessness, did not seek help until he was 25.

Kate (below) had arthritis herself, but at first attributed her daughter Jemima's symptoms to other possible causes despite her own “insider” knowledge about the condition:

I first got diagnosed with arthritis when I was in my early thirties (…) [then] I had two children and it was some time, maybe three or four years after that [my daughter] started complaining of similar problems. Pain with her feet was the first thing which I then put down to possibly shoes not fitting properly or ‘growing pains’.

(Kate, mother of Jemima diagnosed age 10)

As we go on to explore in the next section, participants’ own sense‐making around early signs and symptoms were often mirrored in their interactions with health professionals when they attended medical consultations prior to diagnosis.

#### 2.1.2 Health professionals and candidacy

Age‐related explanations also seemed to mediate interactions with health professionals, who tended to favour other age‐plausible reasons for symptoms, such as “growing pains”:

Mother: But it's also, you take them to the doctors. They say growing pains. She's at that age where she was having growing, you know, when you'd expect them to have growing pains, so you believe what the GP, you know.

Father: It's only when you go on the website and see how many kids it affects then you might put two and two together but, if you're a doctor seeing loads of patients every week, you're not going to associate it with necessarily arthritis here.

(Parents of Kay, diagnosed age 16)

After several clinical encounters with a number of professionals providing multiple rationales explaining away symptoms, arthritis was often not even considered as an option by health professionals. For example,

I did eventually go to my GP. I think I went three or four times before I was actually diagnosed. Again I was turned away, just saying that it was a virus, that there was nothing really that they could do […] but I don't think anyone suspected arthritis or even questioned me along those lines.

(Kim diagnosed age 22)

In other cases, these age‐plausible explanations together with the frequent attendance were compounded by other stereotypical assumptions about young people's behaviour:

I was constantly told I had a sprained ankle. My mum was actually told that I was playing acting even though it was quite obvious [that] my ankle was swollen.

(Jacqueline, diagnosed age 13)

Sometimes, seeing different GPs or attending at emergency departments could cause further delays in diagnosis, with each health professional referring to age‐plausible explanations of symptoms. This lack of continuity could also prevent links being made, for example with family history and the wider context of the person. On the other hand, seeing a different GP with a particular expertise in arthritis in young people eventually led to David's diagnosis:

There wasn't just the one GP. There was a few GPs and as you book appointments, it wasn't really consistent. I was seeing different GPs a lot of the time. So that, to an extent, maybe didn't help because I was getting a follow‐up each time I was having to explain the case definitely every time and that that was that maybe didn't help because every time I went to a new doctor they didn't have the understanding of previous appointments. So I suppose that could have delayed things slightly but I think. In the first GP practice, I must have seen about five to six GPs […]. In the in the new GP practice, I'd only just seen the one because he spotted it straight away because he'd just come back from the conference.

(Daniel, diagnosed age 21)

#### 2.1.3 Negotiating candidacy with health professionals

People with adult‐onset asthma often presented with a persistent cough or recurring chest infections initially. Anita felt progressively more unwell over several months whilst other possible causes, such as the possibility of heart problems, were explored before asthma was considered. In fact, despite a family history of asthma, her doctor did not consider it as possibility until she herself suggested it:

One day I was in such, I had such a lot of chest pain and I just couldn't breathe that I just made myself an emergency appointment and said to her, ‘Look I think it's asthma, I've got this family history, of very severe asthma in several family members […] I'm in such pain, would you not think it appropriate to try and prescribe me some asthma medication and let's just see if that improves my condition’.? So in a sense I diagnosed myself.

(Anita, diagnosed age 53)

Evelyn had experienced what she had been told was hay fever for many years, before eventually putting the idea of asthma to her GP herself:

I had this problem getting up the hill and getting so breathless I couldn't do it, I couldn't get up the hill, and I had to stop, literally. […] I thought wait a second, something's wrong here. And I said to my GP what the problem was and I said, “This isn't asthma is it?” Because I'd had hay fever for over twenty years by then, and she said, “Well it kind of sounds like it is.

(Evelyn, diagnosed age 36)

Likewise, young people with arthritis reported similar experiences where after some time they themselves, or a parent, suggested the diagnosis:

I just [saw] the normal doctor and then they like referred me to a hospital and then it just sort of like spiralled on to different places (…) when I first started getting like my hands were all stiff and stuff, they just looked at it, and my Mum was like, “I think it's arthritis,” and then they were like, “Oh no it isn't.” And then so like I kept going back […]

**Researcher**: So it was your Mum that identified it before the GP?

Yeah, ‘cos she has it as well, so she was like, “Oh I think that might be arthritis.

(Jemima, diagnosed age 10)

Kate, who had been diagnosed with arthritis at the age of 34, explained the similarities she noticed between her daughter Jemima's symptoms and her own:

Maybe a month later, I noticed her writing, she was holding her pen awkwardly and she had her index finger pointed straight and I said to her, “Why, why are you holding your pencil like that? Why are you holding your finger straight?” and she said, “Because I can't bend it,” and I said, “What do you mean you can't bend it?” and she said, “It just won't bend”. So that was when the alarm bells really did ring then and so I looked at her hands and I could see that her, some of her, her joints were slightly swollen on her thumbs. So I really thought, “I think she must have arthritis”. […]. So I took her back to the doctors. He then, after some pressuring, sent her for blood tests; he then referred her after the blood tests to the local hospital to a consultant paediatrician and (…) couldn't really explain it so I then pressed him and said, “Well if you can't explain it, could it be arthritis?” […] they confirmed it. They said it wasn't an obvious form of it […] you know, it wasn't something that screamed out arthritis when they first saw her.

(Kate, mother of Jemima diagnosed age 5)

In the case of asthma, sometimes it was friends, colleagues or family members who first suggested asthma as a potential cause:

A colleague of mine at work suggested that I go and get my breathing checked out, because I was coming up one flight of stairs and was really breathless. And it took me a while to do that, because I was always quite fit and healthy and I didn't think that I had anything wrong with my breathing.

(Veronica, diagnosed age 57)

## 3 DISCUSSION AND CONCLUSION

We have analysed our findings using the concept of “candidacy” defined by Dixon‐Woods et al^5^ as “the ways in which people's eligibility for medical attention and intervention is jointly negotiated between individuals and health services.” We have included patients’ own perspectives including their experiences of interactions with GPs, who seem to draw on similar age‐related understandings. We also draw on Evans et al's^42^ expansion of Anderson et al's^43^ model of “total patient delay,” describing both patient factors affecting help‐seeking, and treatment delays on the part of health professionals and the health system.

Candidacy starts with the perception of symptoms either by the individual, or in the case of a childhood‐onset condition, in collaboration with parents or carers. Essentially symptoms must be perceived to be significant and requiring medical attention. Andersen et al^43^ describe a process of identifying unexplained signs or symptoms, appraising whether these signs constitute “illness,” deciding whether such illness merits medical help, and finally getting round to making an appointment.

As with any medical condition, participants sometimes took a whilst to realize there was anything significant in apparently vague and intermittent symptoms. Even when they appraised their symptoms as illness, they and family members sometimes searched for age‐plausible explanations first, such as growing pains or sports injury in young people with arthritis, or being unfit or just getting older in asthma. Few immediately connected their symptoms with asthma or arthritis—despite several people having clear family examples that might have led them to a faster comparison if they had realized the condition was not bound to a particular age group.

So whilst people might have asserted a generic claim of candidacy as an ill person, or on behalf of an ill child, they typically did not assert candidacy as a person with asthma or arthritis. Exceptionally, some people eventually put two and two together themselves and attempted to negotiate their candidacy more proactively by suggesting a diagnosis to the GP. However, this had mixed results. For example, Anita felt she successfully persuaded her GP to think of asthma as a possibility, whereas Kay's parents reported that the GP “just said no.”

Although the narratives specifically provide patient perspectives, these included their accounts and interpretations of how GPs responded to reported symptoms in similar ways. The narratives provide interesting illustrations of candidacy as a product of the interplay between patient, GP and the wider health system.^44^ Just as individuals may be inclined to seek age‐plausible explanations first, according to these accounts, so too did many GPs. Two of our co‐authors, HS (a GP), and JM (a paediatrician) certainly recognized the picture that our patient narratives paint of such interactions, and the difficulties that can arise for GPs in recognizing and diagnosing these conditions.

Our findings suggest patients perceived GPs in some cases to be minimizing their reported symptoms as insignificant or un‐concerning. Even where symptoms were regarded as worthy of investigation, other diagnoses were often pursued first such as bronchitis, COPD or heart problems in the case of asthma, or muscle and joint trauma in arthritis. Occasionally, a psychological explanation such as attention‐seeking or psychosomatic presentation was regarded as a more age‐plausible explanation in young people than a physical cause. We found a certain resonance here with Evans et al^42^ expansion of Andersen et al's^43^ “treatment delays,” which considered five categories: non‐investigation of symptoms, treatment for other possible causes, lack of follow‐up, referral delays and system delays (such as long waiting times for an outpatient appointment).

As well as barriers to the acceptance of untimely diagnoses by patients, we have also noted that their accounts suggest doctors may be slow to make such diagnoses. It is important to note that, in the UK, GPs ability to pursue particular options may be constrained by protocols which direct the course of investigations and this may well impede their recognition of candidacy. Diagnostic reasoning is informed in part by age plausibility, so for example, a cardiac cause is high on the doctor's list of possibilities in a 60 year old with chest pain, but for a 16 year old, it is very unlikely. This necessary and appropriate step in reasoning may cause error if the doctor has an unrealistic perception of the incidence of a condition at a given age, and doctors may share the lay perception of asthma as primarily a diagnosis that is made in youth. A breathless teenager may be thought likely to have asthma, but a breathless grandparent may have many other cardiac or respiratory causes for their symptoms. Thus, in older people, asthma may only be thought of after other causes of cough and breathlessness have been explored and excluded.

In the case of young people with arthritis, the UK current standards of care^45^ specify that all clinicians and allied health professionals likely to come into contact with a child with JIA (eg GPs, paediatricians, A&E doctors, paediatric physiotherapists) should acquire appropriate clinical skills and knowledge about early recognition of JIA and the need for prompt referral to a paediatric rheumatology team. However, evidence suggests there may be gaps in training in paediatric rheumatology, and sometimes young people are seen initially by clinicians who are not optimally trained or resourced to deliver best practice^46^, ^47^, ^48^, ^49^

As we have seen in some of the examples above, continuity of care may also affect how long it takes to reach a diagnosis. When a patient sees a different doctor each time they attend, there is a tendency for each doctor to start again with the thread of clinical reasoning, and overcoming the perception of age implausibility may take longer overall. Conversely, it is possible for a problem to remain unsolved and just be accepted as normal for that patient after various investigations have revealed no cause for a symptom, and no medications have helped. In this situation, a new doctor bringing fresh eyes and different expertise may be required to make the “untimely” diagnosis.

The diagnosis of both adult‐onset asthma and arthritis in young people may be regarded as an untimely breach of the expected order of things. We have thrown light on the way that patients, and doctors, sometimes draw on age‐related beliefs including “common sense” or “popular” discourses and repertoires as way to make sense, or otherwise, of puzzling symptoms, which can result in delays in diagnosis. We argue here that the experience of being diagnosed with what may be perceived to be a “childhood” condition in adulthood, or an older person's condition in childhood, might be viewed as a “biographical paradox” in that such a diagnosis contradicts common beliefs in relation to what might be seen as usual for a particular age group, and therefore may not be considered initially as an explanation.

Our paper contributes to the sociology of diagnosis, in that we pay particular attention to the diagnosis of chronic illness at a time of life that is seen as dissonant with chronological age. As our findings illustrate, this can have an impact on help‐seeking behaviour, as well as health professionals’ readiness to consider a diagnosis.

### 3.1 Strengths and limitations of the study

The analysis presented here benefited from the knowledge and insights from the original study researchers, as well as clinicians who have experience of diagnosing both conditions. We have used two conditions as exemplars to explore where age‐related expectations have an impact on recognition of symptoms and diagnosis, from the patient perspective, including their experiences of interactions with GPs leading to delays in diagnosis. Interviews with GPs about their experiences of “untimely diagnosis” in these, and potentially other similar conditions such as childhood cancer, would provide valuable additional insights. We have focussed our attention on age‐related barriers, but acknowledge that there are a range of other factors that impact upon health‐seeking behaviours, such as illness awareness and perceptions, and knowledge of the prevalence of these types of conditions.

## CONFLICT OF INTERESTS

The authors declare that they have no competing interests.
